# The Effect of a 10-Week Electromyostimulation Intervention with the StimaWELL 120MTRS System on Multifidus Morphology and Function in Chronic Low Back Pain Patients: A Randomized Controlled Trial

**DOI:** 10.3390/jfmk10040443

**Published:** 2025-11-18

**Authors:** Daniel Wolfe, Brent Rosenstein, Geoffrey Dover, Mathieu Boily, Maryse Fortin

**Affiliations:** 1 Department of Health, Kinesiology, and Applied Physiology, Concordia University, Montreal, QC H4B 1R6, Canadageoffrey.dover@concordia.ca (G.D.); 2 School of Health, Concordia University, Montreal, QC H4B 1R6, Canada; 3 McGill University Health Centre, Montreal, QC H4A 3J1, Canada; 4 Centre de Réadaptation Constance-Lethbridge du CIUSSS COMLT, Montreal, QC H4B 1T3, Canada

**Keywords:** chronic low back pain, EMS, NMES, lumbar multifidus

## Abstract

**Background:** Chronic low back pain (CLBP) patients present with morphological and functional deficits to the lumbar multifidus. Electromyostimulation (EMS) can be used to improve activation and strength in atrophied skeletal muscle, but its effect on multifidus morphology and function in CLBP patients is understudied. The aims of this study were to compare the effect of two EMS protocols on lumbar multifidus morphology, function, and patient-reported outcomes. **Methods:** Two-arm randomized control trial (RCT). Individuals with CLBP were randomized to receive either the ‘phasic’ or ‘combined’ muscle therapy protocol with the StimaWELL 120MTRS, a medium-frequency EMS device. *T*-tests and non-parametric equivalents were used to assess change in imaging-based outcomes, and a repeated-measures ANOVA was used for patient-reported outcomes. Results: Apart from a significant within-group decrease in fatty infiltration at left L5-S1 in the combined group (MD = −1.51, 95% CI = −2.79, −0.23, *p* = 0.024), results revealed no significant within- or between-group changes to multifidus morphology or function. Pairwise comparisons revealed that both groups experienced significant improvements in multiple pain outcome measures, with a significant group*time effect for LBP with sitting (*p* = 0.019) and pain interference (*p* = 0.032) in favor of the phasic group. Additionally, there were significant improvements in the phasic group in disability, pain interference, and pain catastrophizing (all *p* < 0.01). **Conclusions:** A 10-week EMS intervention produced no between-group differences in multifidus muscle morphology or function. Participants in both groups experienced significant improvements in a variety of patient-reported outcomes.

## 1. Introduction

Chronic low back pain (CLBP) affects approximately 20% of the global population [1] and was the leading cause of years-lived with disability in 2020 [2]. CLBP patients present with morphological [3,4] and functional [4] deficits to the lumbar multifidus, a muscle responsible for providing stability to the lumbar spine [5]. At the morphological level, systematic reviews report that patients with CLBP have smaller multifidus cross-sectional area (CSA) at L4 & L5 [6] and elevated multifidus fatty infiltration [3] compared to healthy controls. At the functional level, cross-sectional studies have found a lower contraction ratio for the multifidus at L4 [7] and L5 [4] in patients with CLBP, as well as elevated multifidus stiffness—as assessed with shear-wave elastography (SWE)—in prone resting at L4 [8,9]. One study [10] found that prone stiffness was elevated in CLBP only in the superficial multifidus (SM), which is functionally distinct from the deep multifidus (DM) [11]. This last study also reported that the multifidus contraction ratio (measured with SWE) was reduced in the SM [10].

These aforementioned findings suggest an obvious link between rehabilitation of the lumbar multifidus and a reduction in CLBP. Exercise therapy is a recommended first-line treatment for CLBP [12] and Pilates, strength, and core-based training were all reported to be superior to controls for improving pain and disability in CLBP patients [13]. Furthermore, there is evidence that exercise therapy can increase paraspinal muscle size: a recent RCT from our lab demonstrated that 12 weeks of motor control plus isolated lumbar extension exercises were more effective than general exercise at increasing multifidus CSA at L4-L5 (MD: 0.69 cm^2^, 95% CI: 0.38, 1.00 cm^2^) and L5-S1 (MD: 0.69 cm^2^, 95% CI: 0.39, 1.00 cm^2^) and multifidus thickness at L4 (MD: 0.22 cm^2^, 95% CI: 0.15, 0.29 cm^2^) and L5 (MD: 0.25 cm^2^, 95% CI: 0.18, 0.32 cm^2^) [14]. However, exercise is not always a feasible treatment for CLBP. Individuals with fear-avoidance behaviors may be unwilling to engage or have reduced compliance with regard to exercise [15], while others with reduced mobility and function may find exercise interventions unsustainable, if not impossible. One alternative is electromyostimulation (EMS), a form of transcutaneous electrotherapy designed to stimulate alpha motor neurons, causing involuntary muscular contraction [16]. In particular, medium-frequency electrotherapy (including IFC and pre-modulated IFC) may result in less skin impedance to current, resulting in a more comfortable treatment than lower-frequency alternatives [16,17]. In their narrative review of the effect of IFC therapy, Rampaso and Liebano observed an apparent trade-off between analgesic efficacy and stimulation frequency, with carrier frequencies of 1 kHz producing a greater analgesic effect and pain pressure threshold (PPT) but also greater discomfort than frequencies of ≥4 kHz, while carrier frequencies of 8 and 10 kHz were reported to be less effective at reducing pain, albeit more comfortable [17].

While there is evidence that EMS can affect lower-limb muscle fiber composition and CSA in heterogeneous populations [18], and improve paraspinal strength and endurance in CLBP patients specifically [19,20], its effect on paraspinal morphology and function is under-investigated. The few existing interventional studies have focused on change in resting and contracting multifidus thickness, with conflicting results [21,22,23]. Despite this, there is evidence from cross-sectional studies that exposure to 20 Hz, 50 Hz, and 80 Hz stimulation frequencies all produced significant real-time increases in both superficial multifidus (SM) & deep multifidus (DM) thickness (all *p* < 0.05), with % changes ranging from 12–15% [24]. At the same time, research has neglected other relevant aspects of muscle morphology and function in CLBP, such as muscle stiffness properties and muscle composition. Therefore, the primary aim of this study was to investigate the effects of two 10-week muscle therapy protocols with the StimaWELL 120MTRS system (a medium-frequency electrotherapy device) on multifidus muscle CSA and fat infiltration in CLBP patients. The secondary aims were to investigate their effect on resting multifidus muscle thickness, multifidus contraction, multifidus stiffness, and patient-reported outcomes (pain intensity, pain interference, pain catastrophizing, and disability).

## 2. Materials and Methods

### 2.1. Study Design and Recruitment

**Figure 1:**
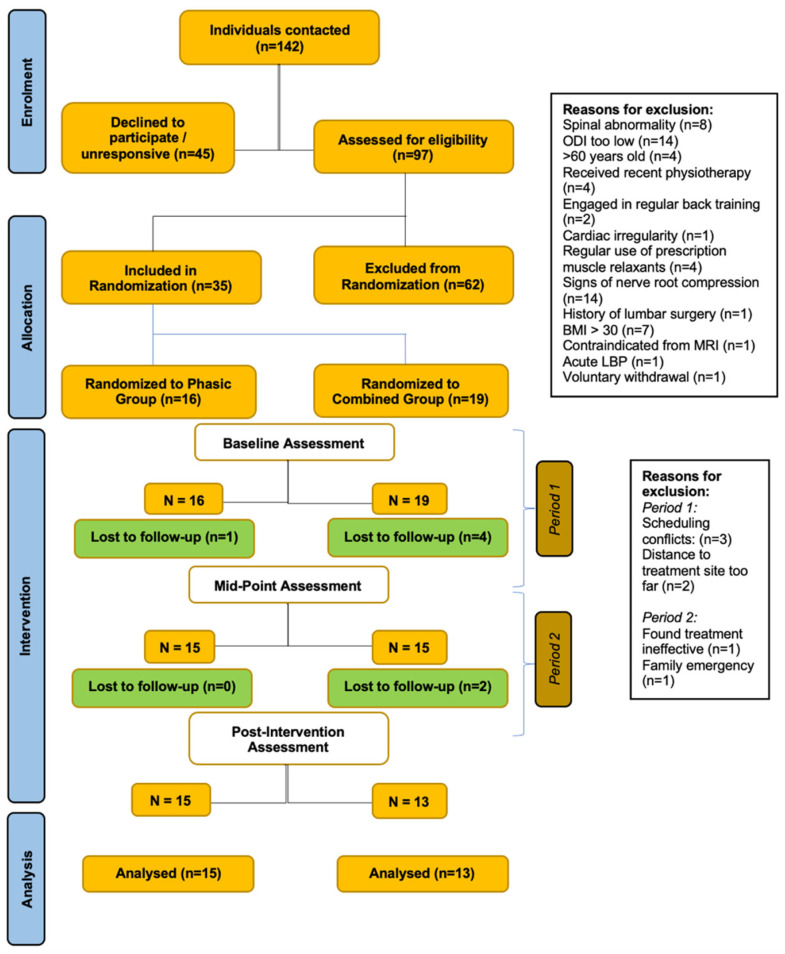
CONSORT Flow Diagram.

This study was a two-arm randomized control trial with test–retest design. We initially recruited 30 participants who were randomized into one of two muscle therapy protocols for the lumbar spine: the ‘phasic’ group or the ‘combined’ group. Due to dropouts that occurred very early due to scheduling issues, we increased the total sample size to 35 participants. The full breakdown of the study design is provided below in the CONSORT flow diagram (Figure 1). The trial was registered at clinicaltrials.gov (registration number: NCT04891682) in May 2021 and a protocol paper was published in July 2022 [25].

Participants were recruited with the help of students and clinicians affiliated with the Quebec Low Back Pain Consortium, through the School of Health (Concordia University, Monreal, QC, Canada) website and mailing list, over social media, and through word-of-mouth. Individuals affiliated with the Quebec Low Back Pain Consortium who agreed to be contacted for studies received either a telephone call or email explaining the study aims and procedure. The recruitment period lasted from January 2022 until October 2024. All prospective participants underwent a preliminary phone screening to verify eligibility. Those who passed the phone screen were invited to the School of Health for a neurological screen, and a trial visit with the StimaWELL 120MTRS system.

### 2.2. Inclusion Criteria

- Chronic non-specific LBP (>3 months), defined as pain in the region between the lower ribs and gluteal folds, with or without leg pain.
- Aged between 18 and 60 years old.
- English or French speakers.
- Have at least score of ‘moderate’ on the Modified Oswestry Disability Index (ODI).
- Able to undergo MRI exam.

### 2.3. Exclusion Criteria

- Currently undergoing or having received physical therapy treatment in the previous month.
- Consistent motor control training for the low back and/or consistent weightlifting, powerlifting, bodybuilding, or strongman training in the previous 6 weeks.
- History of lumbar surgery.
- Presence of positive lumbosacral dermatomes or myotomes.
- Presence of disease which could affect the stiffness of muscle tissue (collagen tissue disease, hemiplegia, multiple sclerosis, blood clots).
- Presence of systemic disease (cancer, metabolic syndrome).
- Presence of spinal abnormality (spinal stenosis, fracture, infection, tumor, or lumbar scoliosis greater than 10 degrees).
- BMI > 30.
- Presence of cardiac arrhythmia.
- Pregnant and breastfeeding women.
- Individuals with epilepsy.
- Individuals at risk of serious bleeding.
- Individuals with pacemakers or metal implants.
- Individuals with aneurysms or heart valve clips.
- Individuals who have taken prescribed muscle relaxants more than once a week in the previous month.

### 2.4. Setting and Randomization

The trial was conducted at the School of Health (Concordia University, Montreal, QC, Canada). The protocol was developed in accordance with the SPIRIT 2013 statement, and was approved by the Central Ethics Research Committee of the Quebec Minister of Health and Social Services (#CCER-20-21-07). All participants provided informed consent prior to beginning the study.

Randomization was achieved using a random computer-generated allocation sequence with permuted blocks, pairing each participant number with a corresponding group (1:1). Group allocations were enclosed in opaque envelopes labelled with numbers. The allocation sequence, participant enrolment, and participant group assignments were carried out by a research assistant who was not involved in administering the intervention or measuring the outcome measures.

A PhD student, who is also a certified athletic therapist, conducted the majority of in-person activities, including enrollment, neurological testing, ultrasound evaluations, and intervention administration. A School of Health technician administered the MRI exam. Participant recruitment and preliminary screening was conducted jointly between the PhD student and the research assistant. Participants remained blinded to group allocation during the entire duration of the intervention. The therapist administering the intervention also served as an assessor, and therefore, could not be blinded to group assignment when collecting outcome measures.

### 2.5. Intervention

All research activities took place at the School of Health (Concordia University, Montreal, QC, Canada). This center houses 8000 m^2^ of laboratories, assessment suites, and lifestyle intervention spaces. It is equipped with the instruments needed to assess this study’s primary outcomes (MRI, Ultrasound), as well as space for conducting the intervention. The intervention period lasted 10 weeks, with treatments occurring twice a week for both groups.

**Figure 2:**
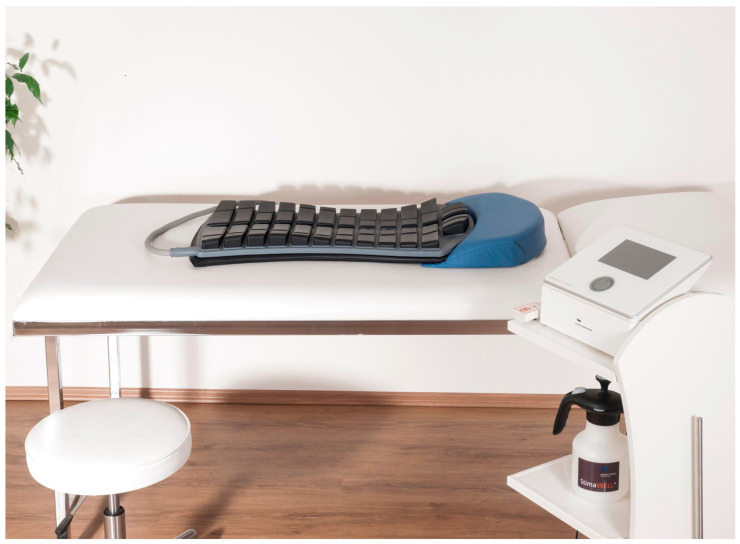
StimaWELL 120MTRS system.

All participants received treatment with the StimaWELL 120MTRS system (schwa-medico, Germany) (Figure 2). The StimaWELL 120MTRS system is a pre-modulated IFC (interferential current) electrotherapy device. It delivers current across up to 12 channels, and offers preset pain and muscle therapy programs. The device also heats up to 40 °C.

Intervention groups: All participants were treated with one of two pre-set muscle therapy programs. Those in the ‘phasic’ group received therapy at the StimaWELL 120MTRS system’s setting for phasic muscle stimulation of the lumbar spine (3 kHz, modulation 50 Hz), which aimed to target mainly Type II muscle fibers. Frequencies of ≥50 Hz produce a tetany response and can generate greater torque than frequencies of <50 Hz, plausibly leading to greater activation of Type II muscle fibers [18]. We chose this setting to try and reverse the apparent shift towards Type I fiber composition in the SM in CLBP patients [10]. Participants in the ‘combined’ group were treated with current delivered at 3 kHz, modulation 4 Hz and 50 Hz. We investigated the efficacy of the combined setting since the majority of multifidus fibers are Type I fibers [26], and low-frequency electrostimulation (<20 Hz) has been reported to increase the percentage of Type I and Type IIa muscle fibers [18]. The combined setting could plausibly induce improvements in both of these fiber types. During the initial calibration and throughout the treatments, the current intensity was increased until participants felt a strong but comfortable contraction. This standard of current intensity was maintained, although the actual output varied over the course of treatment.

Trial visit: Participants who passed the phone screening were invited to the School of Health for a trial visit. After providing informed consent, they underwent a neurological evaluation and filled out a sociodemographic questionnaire. Baseline questionnaires for patient-reported outcomes were also completed at this time. Afterwards, participants received a 10 min trial treatment with the StimaWELL 120MTRS system at its setting for ‘combined’ lumbar muscle therapy.

Wave-mat calibration: The StimaWELL 120MTRS system delivers current at a given intensity across up to 12 channels. However, in the presence of pain or injury the current may not be felt equally across channels. Therefore, prior to the trial, the wave-mat was calibrated to ensure that the current was equally felt across all channels. A paper towel was sprayed with warm water and laid over top of the wave-mat. Participants were asked to remove their top (and unbuckle their bra, if applicable), lie supine on the towel with knees bent, and lower the top of their underwear so that their coccyx was touching the lowest working channel. A blanket was provided for privacy. During the initial phase of calibration, the current travels the length of the wave-mat, from bottom to top, in repetitive fashion. The current was increased to tolerance (i.e., until participants felt a strong, but non-painful, sensation). Then, the current was adjusted, channel by channel (from bottom to top), to ensure that each channel was set to the appropriate intensity. Once complete, the current was adjusted from side-to-side, in groups of two channels (from bottom to top), to ensure the intensity was felt the same from one side to another at a given level. This completes the calibration. This process was repeated prior to the start of participants’ 5th, 9th, 13th, and 17th treatments, to account for unilateral and segmental adaptations, and to ensure the multifidus was appropriately stimulated.

### 2.6. Outcome Measures

MRI assessment of multifidus muscle cross-sectional area: All participants underwent a lumbosacral MRI evaluation using the School of Health’s 3-tesla GE machine in order to assess multifidus muscle CSA and fat infiltration. MR imaging was collected using a standard phased-array body coil with 4 mm slice thickness, 180-mm^2^ field of view and 512 × 512 matrix. Quantitative multifidus muscle measurements were obtained from axial T2-weighted images, bilaterally at the L4-L5 and L5-S1 spinal levels, which are the most relevant levels for spinal pathologies. The multifidus muscle CSA was measured manually at both levels on multiple slices to calculate the summative 3D volume; cross-sectional area measurements have been widely used to assess muscle size and this technique is very reliable (ICCs: 0.97–0.99) [27].

MRI assessment of multifidus muscle fat infiltration: Following the measurement of multifidus CSA, DIXON axial water and fat images were used to assess percent-fat signal fraction at each spinal level according to the following equation: %FSF = (Signal_fat_/[Signal_water_ + Signal_Fat_] × 100) [28].

Ultrasound assessment of multifidus muscle %thickness change during contraction: The School of Health’s Aixplorer ultrasound unit (Supersonic Imagine, Aix-en-Provence, France) was used to assess multifidus muscle contraction and stiffness. First, participants were placed in a prone position, on a therapy table, with a pillow placed under their abdomen to minimize lumbar lordosis (e.g., maximum of 10° measured with an inclinometer) and instructed to relax the paraspinal musculature. The spinous process of L5 was palpated and marked on the skin with a pen prior to imaging. Acoustic coupling gel was applied to the skin and the ultrasound transducer placed longitudinally along the midline of the lumbar spine to confirm the location of the L5 level. The multifidus muscle was imaged bilaterally, in the parasagittal section, allowing for the visualization of the L5/S1 zygapophyseal joints. Multifidus muscle contraction was assessed via contralateral arm lifts while holding a small handheld weight (e.g., 1.5 to 3 pounds) based on the participant’s body weight. Participants were instructed to raise the loaded arm 5 cm off the examination table with the shoulder in 120° of abduction and elbow 90° of flexion, following a deep inhalation and exhalation. Images were taken after the loaded arm was held for 5 s. The handed weight is designed to load the multifidus muscle to approximately 30% of maximal voluntary isometric contraction. Measurements were obtained at L4-L5 and L5-S1 and repeated 3 times, both at rest and during contraction on each side. The average of 3 thickness ratios ([thickness contracted − thickness rest/thickness rest] × 100) was calculated for use in the analysis. This technique is valid and reliable [29,30].

Ultrasound assessment of multifidus muscle stiffness: The same position and procedure was used to assess multifidus muscle stiffness with shear-wave elastography. This technique is based on a compressive wave that propagates within the tissue, allowing for the calculation of tissue shear wave modulus while rendering a quantitative color-coded map of tissue elasticity. Participants lay prone on the therapy table for 5 min before the lumbar multifidus was imaged at rest and during sub-maximal contractions while performing the same task as described above. Three repetitions were performed on each side (both at L4-L5 and L5-S1) and the average shear wave modulus was used for analysis. Finally, multifidus muscle stiffness was examined in a standing position, when the muscle is naturally contracting in a stabilizing role. Participants stood barefoot on the floor with arms relaxed in a natural posture. Resting shear wave modulus measurements were acquired as described above and obtained in the standing fundamental position (e.g., arms resting naturally on each side of the body). Again, three measurements are obtained on each side (both at L4-L5 and L5-S1) and the average shear wave modulus was used in the analysis. This technique is valid and reliable [31,32].

All ultrasound and MRI analysis was performed by the same PhD student who administered the interventin. The student was not blinded to group allocation nor to the time-point of analysis (pre- or post-intervention).

Pain intensity: Pain intensity was measured with the Numerical Pain Rating Scale (NPRS). The NPRS measures pain intensity on a scale of 0–10, with 0 indicated no pain, and 10 indicating the worst pain imaginable. Changes of 2 or more points are clinically significant [31]. At baseline, midpoint, and endpoint, participants were asked to rate the following using the NPRS: current low back pain, current leg pain, best and worst low back pain over the previous week, pain sitting and with movement over the past 24 h. We calculated average low back pain as the average of current pain, and best and worst low back pain over the previous week. Additionally, participants were asked to rate their current low back pain prior to, and at the end of each treatment [33].

Pain interference: Pain interference was measured using the Brief Pain Inventory, interference subsection (BPI). The BPI-I is a 7-item questionnaire that measures how pain interferes with activities of daily living. Each item is rated from 0–10. Higher scores indicate greater interference.

Disability: Disability was assessed with the Oswestry Disability Index (ODI). It is a 10-item scale in which each item is rated from 0–5. Higher scores indicate greater disability, and changes of >10% are clinically significant [34].

Catastrophizing: Pain catastrophizing was assessed with the Pain Catastrophizing Scale (PCS). The PCS is a 13-item questionnaire that assesses the participant’s level of catastrophizing. Each item is rated from 0–4 for a possible total of 52. Higher scores indicate greater catastrophizing, and scores above 30 are clinically significant [35].

### 2.7. Timeline

The intervention period lasted 10 weeks, with treatments occurring twice a week for both groups. There is evidence that a minimum of five weeks of training are needed to induce muscular hypertrophy [36]. The treatment lasted 20 min for the first 3 weeks, 25 min for the second 3 weeks, and 30 min for the last 4 weeks; these times are in line with norms for EMS interventions [37]. Additionally, participants visited the School of Health for two pre-intervention visits (trial visit & questionnaire completion, MRI and ultrasound evaluation), and one post-intervention visit (questionnaire completion, MRI and ultrasound evaluation) for a total of 23 visits (Table 1).

Participants completed the full NPRS, BPI, ODI, and PCS at baseline, midpoint (6 weeks), and post-intervention (11 weeks).

### 2.8. Sample Size Calculation and Statistical Analysis

While the effect of EMS on multifidus muscle morphology and function has not been thoroughly investigated in subjects with CLBP, previous reports showed significant improvements in multifidus thickness at rest and during contraction with medium to large effect sizes following a 6-week [22] and single neuromuscular electrical stimulation (NMES) session [38], respectively. Based on these studies, we used a mean effect size of 0.9 to calculate our sample size at a level of confidence of 0.05 and 80% power. Accordingly, a sample size of 12 participants in each group was needed. We increased the sample size to 15 participants in each group to account for potential drop-out.

An exploratory data analysis was used to verify normality assumptions. Independent *t*-tests and Mann–Whitney-U tests were used to assess between-group changes, pre-to-post intervention, for all MRI and ultrasound measures. Paired *t*-tests and Wilcoxon sign-rank tests were used to assess within-group changes, pre-to-post intervention, for all MRI and ultrasound measures. We used a two-way repeated measures ANOVA to determine changes over time for patient-reported outcome measures. Additionally, after considering the similar nature of both interventions, we decided to pool both groups and assess the overall effect on the intervention on pain-related outcomes using paired *t*-tests. The effect of treatment on current pain (both immediately and over time) was visualized with a line graph. For all tests, statistical significance was set at *p* < 0.05.

## 3. Results

**Figure 3:**
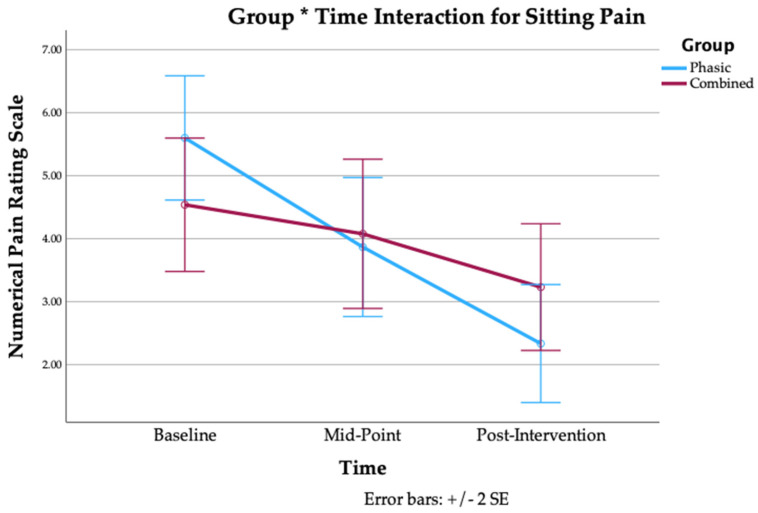
Group * Time Interaction for LBP with Sitting.

**Figure 4:**
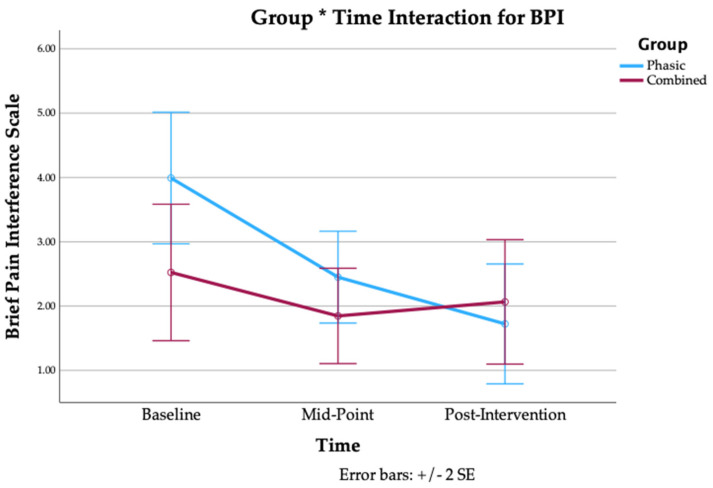
Group * Time Interaction for BPI.

28 participants completed the intervention, 13 in the combined group (average age: 41.0 ± 13.8y; average BMI: 23.7 ± 3.1; average duration of LBP: 133.2 ± 116.2 months) and 15 in the phasic group (average age: 42.0 ± 12.3y; average BMI: 24.9 ± 3.1; average duration of LBP: 79.4 ± 81.9 months). There were no significant between-group baseline differences in sex, age, BMI, or duration of LBP, as reported in Table 1. Our results revealed no between-group differences for any of the morphological outcomes (Table 2 and Table 3) and functional outcomes (Supplementary Tables S1–S5). Additionally, there were no significant within-group changes, with the exception of a significant decrease in fatty infiltration at left L5-S1 in the combined group (MD = −1.51, 95% CI = −2.79, −0.23, *p* = 0.024) (Table 3), and a significant decrease in contraction ratio at right L4-L5 in the combined group (MD = −4.77%, 95% CI = −9.39%, 0.16%, *p* = 0.044) (Table 2). A repeated-measures ANOVA did not reveal a significant main effect of group for any of the patient-reported outcomes. On the other hand, it did show a significant main effect of time on average LBP intensity (*p* < 0.001), LBP with motion (*p* < 0.001), LBP with sitting (*p* < 0.001), disability (*p* < 0.001), pain interference (*p* = 0.003), and pain catastrophizing (*p* = 0.010), with a significant group * time effect for LBP with sitting (*p* = 0.019) and pain interference (*p* = 0.032). The interaction effects are shown below in Figure 3 and Figure 4. No significant main effect of time or interaction effect was found for leg pain intensity (Table 4).

Pairwise comparisons revealed significant improvements in average LBP intensity in both groups from baseline to post-intervention (phasic: *p* < 0.001; combined: *p* < 0.001), from baseline to mid-point (phasic: *p* = 0.004; combined: *p* = 0.021), and from mid-point to post-intervention (phasic: *p* < 0.001; combined: *p* = 0.010). There were also significant improvements to LBP with motion in both groups from baseline to post-intervention (phasic: *p* < 0.001; combined: *p* = 0.005), in the phasic group from mid-point to post-intervention (*p* < 0.001), and in the combined group from baseline to mid-point (*p* = 0.021). There were significant improvements to LBP with sitting in both groups from baseline to post-intervention (phasic: *p* < 0.001; combined: *p* = 0.015), as well as in the phasic group from baseline to mid-point (*p* < 0.001) and from mid-point to post-intervention (*p* < 0.001). The phasic group also experienced significant improvements in leg pain from mid-point to post-intervention (*p* = 0.047). In terms of disability, the phasic group had significant improvements from baseline to post-intervention (*p* < 0.001), from baseline to mid-point (*p* < 0.001), and from mid-point to post-intervention (*p* = 0.018). There were also significant improvements in pain interference in the phasic group from baseline to post-intervention (*p* < 0.001) and from baseline to mid-point (*p* < 0.001). Finally, there were significant improvements in pain catastrophizing in the phasic group from baseline to post-intervention (*p* < 0.004) and from baseline to mid-point (*p* < 0.008). Pairwise comparisons are reported in Table 4.

**Figure 5:**
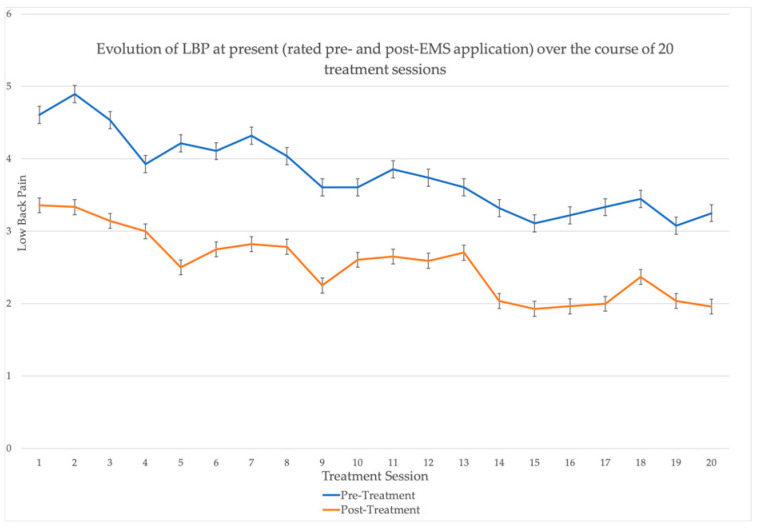
Change in LBP over course of intervention.

With respect to change in pooled average pain intensity from baseline to post-intervention, paired *t*-tests revealed an improvement of −2.059 (95% CI = −2.662, −1.457, *p* < 0.001). Additionally, the changes in pooled pain intensity from baseline to post-intervention (on a per-session basis) are presented below in Figure 5.

### Adverse Events

One participant in the combined group reported a transient increase in numbness at the application site immediately following one treatment; the same participant also reported having a headache immediately following a different treatment. Two other participants in the combined group reported that the current felt less intense on the mat’s bottom right quadrant, while another in the combined group reported that the current felt more pinpointed on the mat’s bottom right quadrant. These issues were corrected via recalibration.

## 4. Discussion

The purpose of our study was to investigate the effect a 10-week EMS intervention with the StimaWELL 120MTRS system on lumbar multifidus morphology and function in patients with CLBP. We also sought to clarify its effect on a variety of patient reported outcomes. Contrary to our hypothesis, our interventions were ineffective at improving multifidus morphology and function. There are a number of possible explanations for these results. Firstly, due to the design of the StimaWELL 120MTRS system, we were unable to target discrete segments of the lumbar multifidus muscle; instead, electrical current was applied to the paraspinal musculature globally. Treatment with the StimaWELL 120MTRS system occurs with participants lying supine, obscuring the precise site of stimulation at a given moment. While the current was increased to tolerance for all participants, who were told that they should be experiencing contractions or pulsations in their lumbar spine, without visibility of the multifidus we cannot guarantee that the intensity of stimulation was high enough to evoke muscle contractions at all times. This may have limited its direct effect on multifidus contraction, especially when considered in light of previous reports [24,28] linking tolerance of higher EMS intensity—likely via a strong depolarizing effect [39]—to greater real-time multifidus thickening. If some participants were unable to tolerate sufficiently higher intensities, the treatment effect would likely be blunted. Secondly, a known drawback of EMS is excessive muscle fatigue [39], since muscle fiber recruitment occurs simultaneously and somewhat randomly [36,39], unlike with voluntary muscle contraction. One way to offset muscle fatigue is with duty-cycles—regular periods of short duration where no current is delivered. However, the preset programs we used in this study do not allow the user to alter the duty cycle; in fact, the programs do not contain any appreciable ‘off’ time. Rather, the programs cycle through two (‘phasic group’) or three (‘combined group’) different pulse patterns during which the four caudal-most channels (that comprise ‘lumbar stimulation’) were activated at varying pulse durations and frequencies. As a result, it is possible that excessive muscle fatigue led to less efficient multifidus force production and a loss of training volume. Lastly, the lack of significant between-group differences was likely due to the similarity of the interventions, as the only difference was the addition of a third pulse pattern in the ‘combined’ group which included 4 Hz modulation.

Existing peer-reviewed research of EMS on lumbar paraspinal muscle morphology and function for CLBP is limited to two studies that assessed muscle thickness. Both studies, one using Russian current [21] and another using Australian current [23] failed to find significant improvements in resting multifidus thickness following 12 20-min treatments. To our knowledge, our study is the first to include MRI and SWE assessments of paraspinal muscle following an EMS intervention for CLBP. On the other hand, the application of EMS for eight weeks in eight healthy individuals aged >65 years old was reported to increase lumbar multifidus CSA by 6.5 ± 9.8% (r = 0.71, *p* = 0.005) on the right side and 6.4 ± 8.2% (r = 0.53, *p* = 0.0036) on the left side, from pre- to post-intervention [40]. Details of how the intervention was implemented (session duration, frequency, and intensity) were not included in the published report, so it is difficult to make a comparison with the other studies cited above or with our trial. It is possible that a greater weekly frequency of sessions is needed to make meaningful changes to paraspinal morphology. Indeed, a report published at the 2011 Annual International Conference of the IEEE Engineering in Medicine and Biology Society, noted that six weeks of once or twice-daily NMES application improved resting right (*p* = 0.0001) and left (*p* = 0.001) lumbar multifidus thickness in CLBP patients [22]. Of note, this study found no significant changes to contracting multifidus thickness. It is also possible that inducing changes to paraspinal muscle morphology and function in CLBP patients requires a different training stimulus than what is needed for healthy individuals.

The lack of morphological changes observed in this study are in agreement with the peer-reviewed reports of similar investigations mentioned above. They also concur with a recent study from our group where we examined the effect of this intervention on erector spinae (ES) morphology from L1-L2 to L5-S1 in a sample of 20 subjects [41]. Findings were very similar: the only significant within-group changes were a decrease in left ES CSA at L2-L3 (MD = −0.35, 95% CI = −0.60, −0.10, *p* = 0.005) and a decrease in left ES %FSF at L5-S1 (MD = −1.65, 95% CI = −3.21, −0.09, *p* = 0.040) (for this analysis both groups were combined). Our analysis revealed a significant decrease in left ES %FSF at L5-S1 in the combined group (MD = −1.51, 95% CI = −2.79, −0.23, *p* = 0.024), and no other significant morphological changes.

As mentioned above (Supplementary Tables S3–S5), the effect of our interventions on multifidus stiffness was minimal. Although this is likely due to a lack of morphological change to the multifidus (as outlined above), it is plausible that our muscle therapy protocols could have reduced resting muscle tone—and therefore stiffness—especially when considered in light of their positive effects on our participants’ pain. After all, does it not seem logical to posit a link between lower pain, a reduced sensation of stiffness, and a reduction in shear modulus? To this point, care must be taken to distinguish between self-reports of muscle stiffness and shear modulus, as the two appear to be independent [42]. An individual might perceive their muscle to be “stiff” without shear modulus being elevated, and vice-versa.

To our knowledge, only one study has investigated the effect of a multi-session intervention on lumbar multifidus stiffness in CLBP patients. Tornblom et al. (2024) [43] assessed the effect of a 12-week motor control plus lumbar strengthening intervention on resting multifidus stiffness at L4 and L5 in CLBP patients. They reported a significant decrease in multifidus stiffness on the right side at L5 (*p* = 0.036), but no change on the left side or at L5, and no change in the contraction ratio either [43]. Considering that an exercise intervention that precisely targeted the lumbar multifidus had a minimal effect on stiffness, it is unsurprising that our results were similarly unimpressive. Although we report a significant decrease in the contraction ratio at right L4-L5 in the phasic group (MD = −4.77%, 95% CI = −9.39%, 0.16%, *p* = 0.044), this is likely due to chance, given the lack of other significant functional findings. Of note, the mean values we found for resting multifidus stiffness at L4 [range: 4.73 ± 1.96, 6.48 ± 3.2] are similar to what has been previously described in the literature [8,10].

With respect to patient-reported outcomes, our results indicate that while both groups experienced similar improvements in pain-based outcome measures, the phasic group experienced greater improvements in disability, pain interference, and pain catastrophizing. However, the pooled improvement in average pain intensity from baseline to post-intervention of −2.059 (95% CI = −2.662, −1.457, *p* < 0.001) matches the cutoff value of Δ2 for clinically important change to the NPRS for CLBP [44]. There were similar improvements (pooled) for pain with motion (MD = −2.123, 95% CI = −2.971, −1.275, *p* < 0.001) and pain with sitting (MD = −2.287, 95% CI = −2.994, −1.580, *p* < 0.001). These improvements in pain are in agreement with previous research into EMS for CLBP [21,23,45]. There are a number of possible explanations for the reduction in pain observed in both groups, including pertinent neurophysiological mechanisms (such as endogenous opioid release and Wedensky inhabitation), and therapeutic alliance (TA). The endogenous opioid response releases enkephalins and endorphins from the periaqueductial grey matter and the rostral ventral medulla in response to small-fiber nerve stimulation [16]. The Wedensky inhibition mechanism describes the physiological interruption of nociceptive impulses from C and A-delta fibers after exposure to stimulation frequencies of >15 Hz and 40 Hz, respectively [17]. The phasic protocol may have induced analgesia via Wedensky inhabitation, while the combined protocol may have additionally reduced pain via the endogenous opioid release system. Additionally, therapeutic alliance may have contributed to treatment efficacy in some cases. While there was no explicit pain education provided to participants, the administrator remained in the room during all treatments, and would engage in conversation with participants when prompted, which may have contributed to the therapeutic effect. Previous research found that sham IFC combined with enhanced TA produced a mean difference of 2.22 cm (95% CI = 18.9–25.0) on the PI-NRS in patients with CLBP, while active IFC combined with limited TA produced a mean difference of 1.83 cm (95% CI = 14.3–20.3), although neither were as effective as active IFC + enhanced TA (mean difference: 3.13 cm; 95% CI = 27.2–33.3) [46]. In our study, a combination of these different factors likely explains the clinically significant reduction in pain that was observed in both groups.

Unlike the combined group, the phasic group experienced significant improvements in disability, pain interference, and pain catastrophizing. From baseline to post-intervention, disability decrease by −10.53% (95% CI = −14.33%, −6.73%, *p* < 0.001), matching three of the six commonly proposed MCIDs described by Schwind et al. (2013): a 30% reduction in disability from initial to final score; a 5–6-point decrease; and a final ODI score of <20% [47]. Pain interference decreased by −2.18 points (95% CI = −3.3, 1.05, *p* < 0.001), approaching the minimum detectable change (MDC) of −2.34 for the BPI-Interference subsection for LBP [48]. Pain catastrophizing decreased by −8 points (95% CI = −13.4, −2.59, *p* < 0.004), exceeding the MDC of 7.73 proposed by Monticone et al. (2022) for individuals with CLBP undergoing multidisciplinary rehabilitation [49], and matching their proposed minimal important change (MIC) of 8 points for individuals with low catastrophizing (PCS < 30) [47]. Considering that the phasic protocol was not superior to the combined protocol at improving muscle characteristics, it is unclear as to why it was more effective for patient-reported outcomes. One possible explanation is that the additional pulse-pattern used in the combined protocol had a negative effect; some patients reported that the relative current intensity during this third pulse-pattern was more intense than during the other two, and this may have led to a less comfortable treatment and less efficient intervention.

Although this is the first study to investigate the effect of the StimaWELL 120MTRS’ EMS protocols, two English-language reports of StimaWELL 120MTRS research have previously been published. In one study [50], 100 patients with non-specific CLBP were randomized into one of four groups: two different dynamic deep-wave protocols with the StimaWELL 120MTRS, a sham electrotherapy protocol with the StimaWELL 120MTRS, and standard care without electrotherapy. Eighteen treatments were given over 6 weeks, and the primary outcome was change in LBP intensity at post-intervention and 12-week follow-up. Results revealed a significant reduction in pain intensity in both deep-wave groups (*p* = 0.000; *p* = 0.000) as well as the sham electrotherapy group at post-intervention (*p* = 0.006), but not in the usual care group. At 12-week follow-up, the improvements were maintained in the deep-wave groups (*p* = 0.001; *p* = 0.001), but not in the sham electrotherapy group (*p* = 0.302). This trial did not specify which parameters were used in the deep-wave groups. More recently, Naka et al. (2023) recruited 54 participants with CLBP or chronic neck pain (CNP) for six 30-min treatments [51]. Participants were randomized into one of three groups: calibration plus treatment (alternating stimulation of 100 Hz and 2 Hz); calibration only; or sham treatment. Study outcomes were trunk range of motion (flexion and extension), pain intensity (NPRS), disability (Neck Disability Index and ODI), and quality-of-life. There were no significant changes in average pain, minimum pain, maximum pain, pain at rest, or pain with activity for participants with either CNP or CLBP in any of the groups. Similarly, there were no significant changes in any group for disability or quality-of-life. However, significant improvements in trunk flexion (*p* = 0.003) and extension (*p* = 0.006) were reported in the group that received electrotherapy. The lack of change to patient-reported outcomes reported by Naka et al. (2023) [51] might be the result of an intervention period of insufficient duration: the results of our study indicate that participants’ LBP improved linearly over an extended period of time (Figure 5).

### Limitations

There were a number of limitations to this study. First, because we lacked a control group, the efficacy of the StimaWELL 120MTRS system could not be compared against other interventions. There is a possibility that some of the improvements in PROMs that were observed were the result of a placebo effect: in previous studies that investigated the effect of a placebo electrotherapy intervention for CLBP, at least one reported statistically significant improvements in pain [52], while others did not [53,54]. However, as previously discussed, a combination of neurophysiological mechanisms and therapeutic alliance are likely to explain the observed gains in PROMs. Second, we decided to schedule treatments twice per week, which is a lower frequency than has typically been used in analogous studies [21,22,23,55]. We took this decision both to maximize participant adherence and to mimic a course of treatment that might be offered with the StimaWELL 120MTRS system in a clinical setting. While many electrotherapy devices are cheap and portable, the design and cost of the StimaWELL 120MTRS makes it less practical for home use than other electrotherapy devices, and this is reflected in our protocol’s relatively low weekly frequency of application. However, this decision may have potentially blunted some treatment effects, particularly vis-à-vis impacts to multifidus morphology and function. Third, due to the design of the StimaWELL 120MTRS system, we were unable to target discrete segments of the lumbar multifidus muscle; rather, electrical current was applied to the paraspinal musculature globally. Treatment with the StimaWELL 120MTRS system occurs with participants lying supine, obscuring the precise site of stimulation at a given moment. While the current was increased to tolerance for all participants, who were told that they should be experiencing contractions or pulsations in their lumbar spine, without visibility of the multifidus we cannot guarantee that the intensity of stimulation was high enough to evoke muscle contractions at all times, which may have generated variability in our participants’ exposure to EMS and limited its direct effect on the multifidus. Fourth, we suffered from a drop-out rate of 20% (7/35), which was higher than anticipated, though only one participant withdrew due to perceived treatment ineffectiveness. Finally, we did not directly assess paraspinal muscle strength or endurance. Although our assessments (which included a battery of self-reported questionnaires and imaging evaluations) were substantial and time-consuming, an evaluation of change to strength or endurance may have provided insight into the discrepancy between our intervention’s limited effect on multifidus morphology and function, and its notable effect on our participants’ self-reported outcomes.

## 5. Conclusions

In sum, 10 weeks of EMS treatment with the StimaWELL 120MTRS system produced no apparent changes to multifidus muscle morphology or function in CLBP patients, apart from a significant reduction in intermuscular fat in the combined group at left L5-SI and a significant reduction in the contraction ratio in the combined group at right L4-L5. However, this finding should be treated with caution considering the absence of a true control group. Both groups experienced significant improvements in pain-based outcomes, while the phasic group additionally benefited from significant reductions in disability, pain interference, and pain catastrophizing. This study adds to the body of evidence that multi-session EMS interventions lead to improvements in PROMS [56], and, given their potential to also improve strength [19] and endurance [20], clinicians should consider prioritizing the use of EMS over sensory forms of electrotherapy such as TENS. Given that research into the effect of EMS on paraspinal morphology and function is an emerging field, future studies should seek to optimize EMS intervention parameters whilst continuing to focus on high-quality outcome measures, such as MRI and SWE.

## Figures and Tables

**Table 1:** Baseline Characteristics (Mean + SD).

	Combined Group (*n* = 13)	Phasic Group (*n* = 15)	Significance
Sex	4 male; 9 female	7 male; 8 female	0.390 ^&^
Age (yrs)	41.0 ± 13.8	42.0 ± 12.3	0.853 ^
BMI	23.7 ± 3.1	24.9 ± 3.1	0.343 ^
Duration of LBP (months)	133.2 ± 116.2	79.4 ± 81.9	0.164 ^

^&^ denotes Pearson Chi-Square test; ^ denotes independent *t*-test.

**Table 2:** Cross Sectional Area (CSA).

	Combined Group (*n* = 13)	Phasic Group (*n* = 15)	Between-Group Difference (Combined Minus Phasic)
L4 level			
Right Pre	8.25 ± 1.77	9.56 ± 2.24	0.51 [−0.15, 1.18], *p* = 0.123 ^ 0.586
Right Post	8.74 ± 1.50	9.54 ± 2.32	
*Difference (post-pre)*	0.49 [−0.05, 1.03], *p* = 0.072 ^a^	−0.02 [−0.47, 0.42], *p* = 0.907 ^a^	
*Effect Size (Hedge’s g)*	0.513	−0.029	
Left Pre	8.38 ± 1.38	9.50 ± 1.89	NA, *p* = 0.316 ^#^
Left Post	8.71 ± 1.43	9.37 ± 1.8	
*Difference (post-pre)*	0.33 [−0.31, 0.98] *p* = 0.221 ^$^	−0.13 [−0.51, 0.25], *p* = 0.472 ^a^	
*Effect Size (Hedge’s g)*	NA	−0.181	
L5 level			
Right Pre	10.28 ± 1.59	11.32 ± 2.85	NA, *p* = 0.294 ^#^
Right Post	10.44 ± 1.75	11.05 ± 2.56	
*Difference (post-pre)*	0.15 [−0.22, 0.54], *p* = 0.463 ^$^	−0.27 [−0.72, 0.17], *p* = 0.213 ^a^	
*Effect Size (Hedge’s g)*	NA	−0.318	
Left Pre	10.39 ± 2.09	11.20 ± 2.36	NA, *p* = 0.217 ^#^
Left Post	10.53 ± 2.10, *p* = 0.256	10.95 ± 2.22	
*Difference (post-pre)*	0.14 [−0.11, 0.39], *p* = 0.256 ^a^	−0.25 [−0.73, 0.23], *p* = 0.363 ^$^	
*Effect Size (Hedge’s g)*	0.310	NA	

^a^ denotes paired *t*-test; ^ denotes independent *t*-test; ^$^ denotes Wilcoxon Sign-Rank Test; ^#^ denotes Mann–Whitney U test.

**Table 3:** %Fat Signal Fraction (FSF).

	Combined Group (*n* = 13)	Phasic Group (*n* = 15)	Between-Group Difference (Combined Minus Phasic)
L4 level			
Right Pre	20.22 ± 9.26	21.84 ± 8.9	NA, *p* = 0.717 ^#^
Right Post	20.18 ± 8.60	21.96 ± 9.49	
*Difference (post-pre)*	−0.03 [−1.51, 1.43], *p* = 0.422 ^$^	0.11 [−0.88, 1.11], *p* = 0.804 ^a^	
*Effect Size (Hedge’s g)*	NA	0.062	
Left Pre	21.48 ± 10.82	23.40 ± 8.82	−2.05 [−4.12, 0.15], *p* = 0.052 ^ −0.750
Left Post	19.82 ± 9.08	23.79 ± 8.97	
*Difference (post-pre)*	−1.65 [−3.46, 0.15], *p* = 0.069 ^a^	0.39 [−0.89, 1.69], *p* = 0.520 ^a^	
*Effect Size (Hedge’s g)*	−0.518	0.161	
L5 level			
Right Pre	24.86 ± 10.29	26.34 ± 9.93	NA, *p* = 0.586 ^#^
Right Post	24.10 ± 9.59	25.94 ± 10.31	
*Difference (post-pre)*	−0.76 [−2.02, 0.49], *p* = 0.210 ^a^	−0.39 [−2.36, 1.56], *p* = 0.394 ^$^	
*Effect Size (Hedge’s g)*	−0.343	NA	
Left Pre	24.55 ± 11.01	27.11 ± 8.23	NA, *p* = 0.170 ^#^
Left Post	23.04 ± 9.43	26.89 ± 8.73	
*Difference (post-pre)*	−1.51 [−2.79, −0.23], ***p***** = 0.024** ^a^	−0.21 [−1.23, 0.79], *p* = 0.363 ^$^	
*Effect Size (Hedge’s g)*	−0.669	NA	

^a^ denotes paired *t*-test; ^ denotes independent *t*-test; ^$^ denotes Wilcoxon Sign-Rank Test; ^#^ denotes Mann–Whitney U test; Bold denotes statistical significance (*p* < 0.05).

**Table 4:** Secondary Outcomes.

Variable	Measurement Timepoint	Phasic Group (*n* = 15)	Combined Group (*n* = 13)	Main Effect of Group	Interaction Effect of Group * Time
Average LBP	*Baseline*	4.97 ± 1.05	4.97 ± 1.51	F = 0.477, *p* = 0.496, df = 1 η^2^ = 0.018	F = 0.971, *p* = 0.385, df = 2 η^2^ = 0.036
	*6-week*	3.79 ± 1.31 **^y^**	4.05 ± 1.54 **^y^**		
	*11-week*	2.57 ± 0.93 **^xz^**	3.25 ± 1.58 **^xz^**		
	*Main Effect of Time*	F = 19.522, *p* < 0.001, df = 2 η^2^ = 0.610	F = 8.115, *p* = 0.002, df = 2 η^2^ = 0.394		
LBP with Motion	*Baseline*	4.46 ± 2.29	4.61 ± 1.85	F = 0.126, *p* = 0.725, df = 1 η^2^ = 0.005	F = 0.875, *p* = 0.396, df = 1.482 η^2^ = 0.033
	*6-week*	3.40 ± 1.99	3.15 ± 1.99 **^y^**		
	*11-week*	2.06 ± 1.38 **^xz^**	2.76 ± 1.42 **^x^**		
	*Main Effect of Time*	F = 13.395, *p* < 0.001, df = 2 η^2^ = 0.517	F = 4.517, *p* = 0.021, df = 2 η^2^ = 0.265		
LBP with Sitting	*Baseline*	5.60 ± 1.54	4.53 ± 2.25	F = 0.001, *p* = 0.981, df = 1 η^2^ = 0.000	F = 4.271, *p* = 0.019, df = 2 η^2^ = 0.141
	*6-week*	3.86 ± 2.06 **^y^**	4.07 ± 2.21		
	*11-week*	2.33 ± 1.44 **^xz^**	3.23 ± 2.16 **^x^**		
	*Main Effect of Time*	F = 24.411, *p* < 0.001, df = 2 η^2^ = 0.661	F = 3.424, *p* = 0.056, df = 2 η^2^ = 0.206		
Leg Pain	*Baseline *	2.46 ± 2.61	1.07 ± 2.01	F = 1.992, *p* = 0.170, df = 1 η^2^ = 0.071	F = 1.809, *p* = 0.174, df = 2 η^2^ = 0.065
	*6-week*	2.46 ± 2.72	1.0 ± 1.35		
	*11-week*	1.53 ± 2.03 ^z^	1.38 ± 2.14		
	*Main Effect of Time*	F = 2.334, *p* = 0.118, df = 2 η^2^ = 0.157	F = 0.317, *p* = 0.731, df = 2 η^2^ = 0.025		
ODI (%)	*Baseline*	26.27 ± 8.13	26 ± 6.92	F = 1.363, *p* = 0.254, df = 1 η^2^ = 0.050	F = 2.740, *p* = 0.092, df = 1.46 η^2^ = 0.095
	*6-week*	19.93 ± 9.46 **^y^**	24.31 ± 5.93		
	*11-week*	15.73 ± 10.4 **^xz^**9	21.31 ± 7.697		
	*Main Effect of Time*	F = 12.459, p *<* 0.001, df = 2 η^2^ = 0.499	F = 1.817, *p* = 0.183, df = 2 η^2^ = 0.127		
BPI	*Baseline*	3.99 ± 2.05	2.52 ± 1.75	F = 1.167, *p* = 0.29, df = 1 η^2^ = 0.045	F = 4.054, *p* = 0.032, df = 1.617 η^2^ = 0.140
	*6-week*	2.45 ± 1.44 **^y^**	1.84 ± 1.20		
	*11-week*	1.72 ±1.49 **^x^**5	2.06 ± 1.976		
	*Main Effect of Time*	F = 9.221, *p* = 0.001, df = 2 η^2^ = 0.435	F = 1.412, *p* = 0.26, df = 2 η^2^ = 0.106		
PCS	*Baseline*	17.73 ± 11.39	18.3 ± 10.32	F = 0.740, *p* = 0.397, df = 1 η^2^ = 0.028	F = 0.823, *p* = 0.403, df = 1.327 η^2^ = 0.031
	*6-week*	11.33 ± 8.15 **^y^**	15.38 ± 11.14		
	*11-week*	9.73 ± 7.39 **^x^**	13.61 ± 10.44		
	*Main Effect of Time*	F = 4.685, *p* = 0.019, df = 2 η^2^ = 0.273	F = 1.603, *p* = 0.221, df = 2 η^2^ = 0.114		

Pairwise comparisons: **^x^** denotes significant difference from baseline to post-intervention; **^y^** denotes significant difference from baseline to mid-point; **^z^** denotes difference from baseline to post-intervention (all *p* < 0.05).

## Data Availability

The original contributions presented in this study are included in the article/supplementary material. Further inquiries can be directed to the corresponding author.

## Supplementary Materials

The following supporting information can be downloaded at: https://www.mdpi.com/article/10.3390/jfmk10040443/s1.
