# Supplementary material for: The Effect of a 10-Week Electromyostimulation Intervention with the StimaWELL 120MTRS System on Multifidus Morphology and Function in Chronic Low Back Pain Patients: A Randomized Controlled Trial
Source: J Funct Morphol Kinesiol. 2025 Nov 18;10(4):443. doi: 10.3390/jfmk10040443 (PMC12641961; doi:10.3390/jfmk10040443)
Supplement: Supplementary file 1 [file jfmk-10-00443-s001.zip › jfmk-3915634-supplementary.pdf]

Supplementary Table S1. Multifidus Prone Thickness (Rest)

	**Combined Group (n=13)**	**Phasic Group (n=14)**	**Between-group Difference (combined minus phasic)**
L4 level			
Right Pre	2.81 ± 0.38	2.97 ± 0.47	
Right Post	2.87 ± 0.36	2.99 ± 0.42	
Difference (post-pre)	0.05 [-0.05, 0.16], p=0.274[a]	0.02 [-0.15, 0.18] p=0.823[a]	0.04 [-0.15, 0.23] p=0.675^
Effect Size (Hedge's g)	0.298	-0.057	0.159
Left Pre	2.88 ± 0.30	2.94 ± 0.44	
Left Post	2.92 ± 0.30	2.98 ± 0.47	
Difference (post-pre)	0.03 [-0.07, 0.15], p=0.463[a]	0.04 [-0.11, 0.19] p=0.596[a]	0.01 [-0.19 0.19] p=0.991^
Effect Size (Hedge's g)	0.204	-0.137	0.004
L5 level			
Right Pre	2.75 ± 0.44	2.92 ± 0.55	
Right Post	2.75 ± 0.39	2.92 ± 0.53	
Difference (post-pre)	0.00 [-0.11, 0.11], p=0.463[$]	0.00 [-0.19, 0.21] p=0.551[$]	NA, p=1.0[#]
Effect Size (Hedge's g)	NA	NA	
Left Pre	2.80 ± 0.25	2.89 ± 0.52	
Left Post	2.82 ± 0.35	2.90 ± 0.50	
Difference (post-pre)	0.02 [-0.12, 0.17], p=0.774[a]	0.01 [-0.14, 0.16] p=0.909[a]	0.01 [-0.19, 0.21] p=0.904^
Effect Size (Hedge's g)	0.076	-0.029	0.046

[a]denotes paired t-test; ^denotes independent t-test; [$]denotes Wilcoxon Sign-Rank Test; [#]denotes Mann-Whitney U test

Supplementary Table S2. Multifidus Prone Contraction Ratio (%)

	**Combined Group (n=13)**	**Phasic Group (n=14)**	**Between-group Difference (combined minus phasic)**
L4 level			
Right Pre	15.34 ± 8.63	12.50 ± 11.68	
Right Post	10.57 ± 6.95	12.64 ± 7.3	

Difference (post-pre)<br><br>*Effect Size (Hedge's g)*	-4.77 [-9.39, -0.16]<br>**p=0.044[a]**<br><br>0.586	0.14 [-6.53, 6.82]<br>p=0.468[$]<br><br>NA	NA, p=0.105[#]
Left Pre	15.04 ± 8.59	15.64 ± 8.12	
Left Post	13.30 ± 5.76	15.21 ± 6.14	
Difference (post-pre)<br><br>*Effect Size (Hedge's g)*	-1.73 [-6.94, 3.48],<br>p=0.481[a]<br><br>0.196	-0.49 [-5.75, 4.89]<br>p=0.865[a]<br><br>-0.044	-1.23 [-8.3, 5.84]<br>p=0.723^<br><br>-0.137
L5 level			
Right Pre	7.90 ± 7.92	5.21 ± 7.01	
Right Post	5.24 ± 6.84	7.57 ± 6.40	
Difference (post-pre)<br><br>*Effect Size (Hedge's g)*	-2.65 [-8.10, 2.79]<br>p=0.309[a]<br><br>0.276	2.35 [-3.25, 7.96]<br>p=0.381[a]<br><br>0.228	-4.79 [-12.25, 2.65]<br>p=0.197^<br><br>-0.495
Left Pre	10.81 ± 4.41	10.57 ± 8.82	
Left Post	9.21 ± 7.57	11.21 ± 7.19	
Difference (post-pre)<br><br>*Effect Size (Hedge's g)*	-1.59 [-6.33, 3.13],<br>p=0.476[a]<br><br>0.191	0.64 [-5.00, 6.28]<br>p=0.810[a]<br><br>0.062	-2.31 [-9.40, 4.77]<br>p=0.507^<br><br>-0.984

[a]denotes paired t-test; ^denotes independent t-test; [$]denotes Wilcoxon Sign-Rank Test; [#]denotes Mann-Whitney U test; **Bold** denotes statistical significance (p < 0.05)

Supplementary Table S3. Multifidus Prone Stiffness (Rest)

	**Combined Group (n=13)**	**Phasic Group (n=14)**	**Between-group Difference (combined minus phasic)**
L4 level			
Right Pre	4.73 ± 1.96	5.08 ± 2.45	
Right Post	5.64 ± 5.71	5.39 ± 3.28	
Difference (post-pre)<br><br>*Effect Size (Hedge's g)*	0.90 [-2.36, 4.18],<br>p=0.507[$]<br><br>NA	0.31 [-1.15, 1.78],<br>p=0.654[a]<br><br>0.115	NA, p=0.402[#]
Left Pre	6.48 ± 3.2	5.99 ± 2.82	
Left Post	5.60 ± 2.06	5.46 ± 2.66	
Difference (post-pre)	-0.87 [-2.48, 0.72],<br>p=0.254[a]	-0.52 [-1.67, 0.62],<br>p=0.34[a]	-0.35 [-2.18, 1.47],<br>p=0.694^

Effect Size (Hedge's g)	-0.323	-0.249	-0.152
L5 level			
Right Pre	4.52 ± 1.22	6.07 ± 5.04	NA, p=0.430[#]
Right Post	5.59 ± 4.05	4.96 ± 2.99	
Difference (post-pre)<br><br>*Effect Size (Hedge's g)*	1.06 [-1.32, 3.45], p=0.917[$]<br><br>NA	-1.11 [-2.93, 0.70], p=0.331[$]<br><br>NA	
Left Pre	4.38 ± 1.59	5.54 ± 2.94	NA, p=0.128[#]
Left Post	4.80 ± 2.60	4.87 ± 2.14	
Difference (post-pre)<br><br>*Effect Size (Hedge's g)*	0.42 [-0.53, 1.39], p=0.422[$]<br><br>NA	-0.67 [-1.88, 0.52], p p=0.244[a]<br><br>-0.307	

[a]denotes paired t-test; ^denotes independent t-test; [$]denotes Wilcoxon Sign-Rank Test; [#]denotes Mann-Whitney U test

Supplementary Table S4. Multifidus Prone Stiffness (Contracted)

	**Combined Group (n=13)**	**Phasic Group (n=14)**	**Between-group Difference (combined minus phasic)**
L4 level			
Right Pre	15.43 ± 8.69	14.15 ± 5.83	0.08 [-5.55, 5.73], p=0.974^<br><br>0.012
Right Post	15.27 ± 6.98	13.91 ± 6.23	
Difference (post-pre)<br><br>*Effect Size (Hedge's g)*	-0.15 [-4.44, 4.13], p=0.938[a]<br><br>-.021	-0.24 [-4.38, 3.89], p=0.899[a]<br><br>-0.034	
Left Pre	15.88 ± 7.96	15.54 ± 6.43	0.24 [-3.88, 4.37], p=0.904^<br><br>0.047
Left Post	14.23 ± 5.18	16.95 ± 9.29	
Difference (post-pre)<br><br>*Effect Size (Hedge's g)*	1.64 [-1.39, 4.68], p=0.258[a]<br><br>0.320	1.40 [-1.72, 4.52], p=0.348[a]<br><br>0.253	
L5 level			
Right Pre	15.59 ± 6.86	13.12 ± 4.51	-0.56 [-6.49, 5.37], p=0.847^
Right Post	16.13 ± 10.28	14.22 ± 5.04	
Difference (post-pre)	0.53 [-4.66, 5.74], p=0.825[a]	1.10 [-2.38, 4.59], p=0.505[a]	

Effect Size (Hedge's g)	0.059	0.178	-0.074
Left Pre	19.70 ± 9.63	21.16 ± 7.2	-2.20 [-9.54, 5.13], p=0.541^<br><br>-0.236
Left Post	15.33 ± 6.19	19.00 ± 12.09	
Difference (post-pre)<br><br>*Effect Size (Hedge's g)*	-4.36 [-9.47, 0.74], p=0.087[a]<br><br>-0.483	-2.15 [-7.98, 3.66], p=0.435[a]<br><br>-0.210	

[a]denotes paired t-test; ^denotes independent t-test

Supplementary Table S5. Multifidus Standing Stiffness

	**Combined Group (n=13)**	**Phasic Group (n=14)**	**Between-group Difference (combined minus phasic)**
L4 level			
Right Pre	12.75 ± 8.54	14.44 ± 8.38	0.41 [-3.32, 4.16], p=0.820^<br><br>0.086
Right Post	11.21 ± 6.35	12.49 ± 6.94	
Difference (post-pre)<br><br>*Effect Size (Hedge's g)*	-1.53 [-4.57, 1.51], p=0.295[a]<br><br>-0.285	-1.95 [-4.50, 0.59], p=0.122[a]<br><br>-0.416	
Left Pre	13.21 ± 5.26	12.31 ± 9.24	1.33 [-3.35, 6.02], p=0.562^<br><br>0.224
Left Post	12.87 ± 6.23	11.31 ± 8.06	
Difference (post-pre)<br><br>*Effect Size (Hedge's g)*	0.34 [-2.87, 3.55], p=0.819[a]<br><br>0.063	-0.99 [-4.63, 2.65], p=0.567[a]<br><br>-0.148	
L5 level			
Right Pre	14.78 ± 10.35	14.01 ± 11.34	0.34 [-5.27, 5.95], p=0.901^<br><br>0.047
Right Post	14.78 ± 10.33	13.67 ± 9.18	
Difference (post-pre)<br><br>*Effect Size (Hedge's g)*	0.00 [-5.42, 5.43], p=0.999[a]<br><br>0.00	-0.33 [-3.03, 2.35], p=0.790[a]<br><br>-0.068	
Left Pre	17.06 ± 7.37	14.12 ± 11.02	1.51 [-6.63, 3.6], p=0.547^<br><br>-0.228
Left Post	14.79 ± 6.89	13.37 ± 11.40	
Difference (post-pre)<br><br>*Effect Size (Hedge's g)*	-2.26 [-5.83, 1.29], p=0.191<br><br>-0.360	-0.75 [-4.75, 3.25], p=0.692[a]<br><br>-0.102	

[a]denotes paired t-test; ^denotes independent t-test
